# A56 DEVELOPMENT OF A PROGNOSTIC SURVIVAL MODEL FOR PATIENTS DIAGNOSED WITH PANCREATIC CANCER IN ONTARIO

**DOI:** 10.1093/jcag/gwac036.056

**Published:** 2023-03-07

**Authors:** P James, A Gayowsky, M Salim, H Seow, R Sutradhar, P Tanuseputro, N Coburn, J Hallet, A Hsu, A Mahar, C Webber

**Affiliations:** 1 Gastroenterology, University Health Network; 2 Medicine, University of Toronto; 3 Institute for Clinical Evaluative Sciences, Toronto; 4 Oncology, McMaster, Hamilton; 5 Department of Biostatistics, University of Toronto, Toronto; 6 Ottawa Hospital Research Institute, Ottawa; 7 Sunnybrook Health Sciences Centre, Toronto; 8 Bruyère Research Institute , Ottawa; 9 Queens University, Kingston, Canada

## Abstract

**Background:**

Pancreatic adenocarcinoma (PAC) is a deadly disease with an overall 5-year survival of less than 8%. The current literature on patient outcomes are limited by small samples sizes and patients enrolled in clinical trials. There are no prognostic tools for patients with pancreatic cancer.

**Purpose:**

To develop a prognostic survival model for patients with pancreatic cancer

**Method:**

All patients with a diagnosis of pancreatic cancer cancer from January 2007 to December 2020 were identified through the Ontario Cancer Registry. The primary outcome was survival. The cohort was used to develop a multivariable cox proportional hazards regression model with baseline characteristics under a backward stepwise variable selection process to predict the risk of mortality. Covariates included patient age, sex, tumour location, cancer stage, treatment types, distance to a cancer centre, hospitalizations, comorbidities, access to family physician, and symptoms as captured using the Edmonton Symptom Assessment System datasets.

**Result(s):**

There was a total of 17,450 pancreatic cancer patients in the cohort, 48% of which were female and the mean age was 72 years. 44% of patients presented with a tumor in the head of the pancreas. Among those with stage data (44%), 24% were stage IV at diagnosis. Mean survival was approximately 0.7 years. Approximately 60% were hospitalized in the 3 months prior to diagnosis. Almost all patients had a family doctor rostered (95%). In multivariate analysis, key predictors of survival assessed at the time of diagnosis were age, sex, tumour location in the pancreas, stage at diagnosis, pain, appetite functional status and treatment choice (all p<0.001). Using these variables, we created a prediction model that can estimate one-year probability of death with high discrimination (area under the curve = 0.82, c-statistic 0.76).

**Conclusion(s):**

Our model accurately predicts one-year pancreatic cancer survival risk using clinical symptom and performance status data. The model has the potential to be a useful prognostic tool that can be completed by patients and their caregivers in support of patient-centered care.

**Please acknowledge all funding agencies by checking the applicable boxes below:**

CIHR

**Disclosure of Interest:**

None Declared

